# A novel xylogenic suspension culture model for exploring lignification in *Phyllostachys* bamboo

**DOI:** 10.1186/1746-4811-8-40

**Published:** 2012-09-14

**Authors:** Shinjiro Ogita, Taiji Nomura, Takao Kishimoto, Yasuo Kato

**Affiliations:** 1 Department of Biotechnology, Toyama Prefectural University, 5180 Kurokawa, Imizu, Toyama, 939-0398, Japan

**Keywords:** Bamboo, Lignification, *Phyllostachys*, Xylogenic suspension culture

## Abstract

**Background:**

Some prominent cultured plant cell lines, such as the BY-2 cell line of tobacco (*Nicotiana tabacum* cv. ‘Bright Yellow 2’) and the T87 cell line of Arabidopsis (*Arabidopsis thaliana* L. Heynh., ecotype Columbia) are used as model plant cells. These suspension cell culture systems are highly applicable for investigating various aspects of plant cell biology. However, no such prominent cultured cell lines exist in bamboo species.

**Results:**

We standardized a novel xylogenic suspension culture model in order to unveil the process of lignification in living bamboo cells. Initial signs of lignin deposition were able to be observed by a positive phloroglucinol-HCl reaction at day 3 to 5 under lignification conditions (LG), i.e., modified half-strength Murashige and Skoog medium (m1/2MS) containing 10 μM 6-benzyladenine (BA) and 3% sucrose. Two types of xylogenic differentiation, both fiber-like elements (FLEs) with cell wall thickening and tracheary elements (TEs) with formation of perforations in the cell wall, were observed under these conditions. The suspension cells rapidly formed secondary cell wall components that were highly lignified, making up approximately 25% of the cells on a dry weight basis within 2 weeks. Detailed features involved in cell growth, differentiation and death during lignification were characterized by laser scanning microscopic imaging. Changes in transcript levels of xylogenesis-related genes were assessed by RT-PCR, which showed that the transcription of key genes like *PAL1*, *C4H*, *CCoAOMT*, and *CCR* was induced at day 4 under LG conditions. Furthermore, interunit linkage of lignins was compared between mature bamboo culms and xylogenic suspension cells by heteronuclear single quantum coherence (HSQC) NMR spectroscopy. The presence of the most common interunit linkages, including β-aryl ether (β-*O*-4), phenylcoumaran (β-5) and resinol (β-β) structures was identified in the bamboo cultured cell lignin (BCCL) by HSQC NMR. In addition to these common features of lignin, several differences in lignin substructures were also found between the BCCL and the bamboo milled wood lignin (BMWL).

**Conclusions:**

Our xylogenic suspension culture model could be used for detailed characterization of physiological and molecular biological events in living bamboo cells.

## Background

Bamboos are a woody monocotyledonous plant species that show unique growth features, especially in culms. They can grow to maturity, up to ca. 20 m high, within a few months due to the expansion of intercalary meristem regions around the upper side of individual nodes. Furthermore, the complexity and diversity of fiber cells in vascular bundles and parenchyma cells in ground tissues are unique in bamboo, as shown in Additional file 1: Figure S1.

Substantial information on the anatomical, physical, chemical and mechanical properties of bamboo culms has been reported. Bamboo fiber and parenchyma cells typically show multilayered cell walls [1-3]. The variability and diversity of fiber and parenchyma cells have also been characterized [4,5]. Changes in levels of chemical components, such as sugars, amino acids, cellulose and lignin, have been analyzed during growth [6-8]. Furthermore, mechanical properties of bamboo materials such as laminated strips have been evaluated [9,10]. However, it is difficult to elucidate detailed information about the biology of living bamboo cells due to the size, growth features and life cycle of bamboo plants grown in a field environment.

Optimization of a cell culture system is a useful and important approach for understanding sequential biological processes in plants of interest. Previously, we established an efficient callus and suspension culture system of *Phyllostachys* bamboo [11] and found that the suspension cultured cells proliferated with highly synchronous morphological features. The system is applicable to investigations of carbohydrate metabolism involved in growth and cell wall development of bamboo cells [12]. We also established transformation protocols using particle bombardment in suspension cell culture systems of *Phyllostachys* bamboo [13,14]. We have now standardized a novel xylogenic suspension culture model in order to unveil the sequential biological steps in lignification of living bamboo cells. In this study, the technical details of the xylogenic suspension culture system and its utility are discussed.

## Results

### Lignification capacity of bamboo suspension cells during subculture

**Figure 1:**
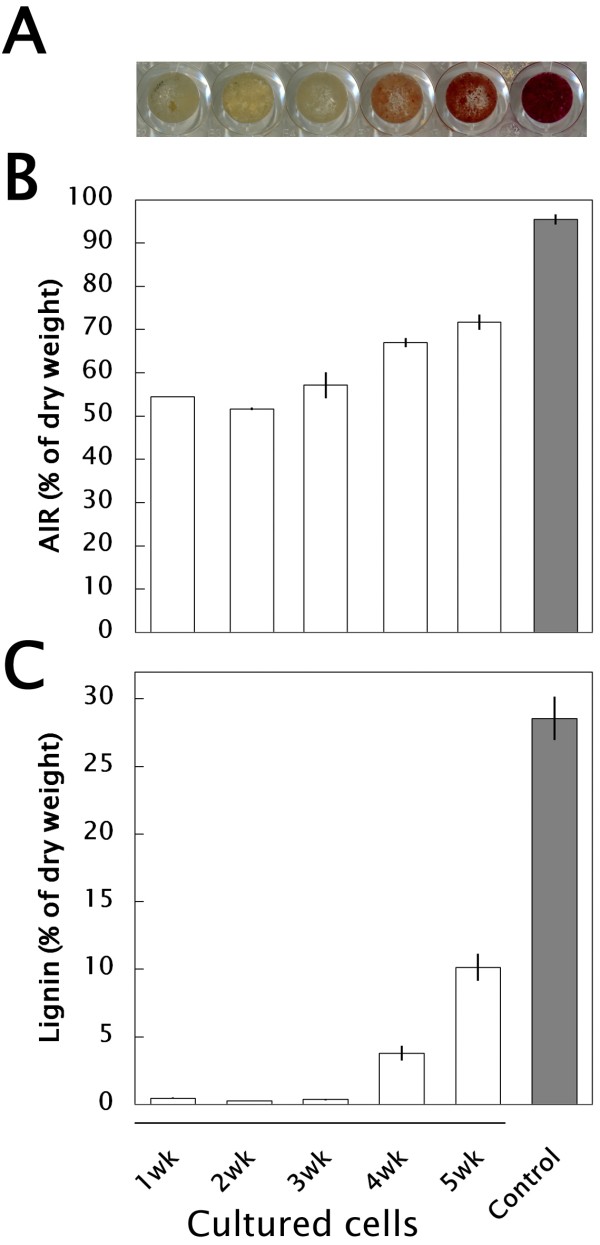
**Lignification capacity of suspension cultured bamboo cells under SC conditions.** (**A**) images of phloroglucinol-HCl reaction. (**B**) AIR content. (**C**) lignin content. Control: mature culm of *P. nigra*.

**Figure 2:**
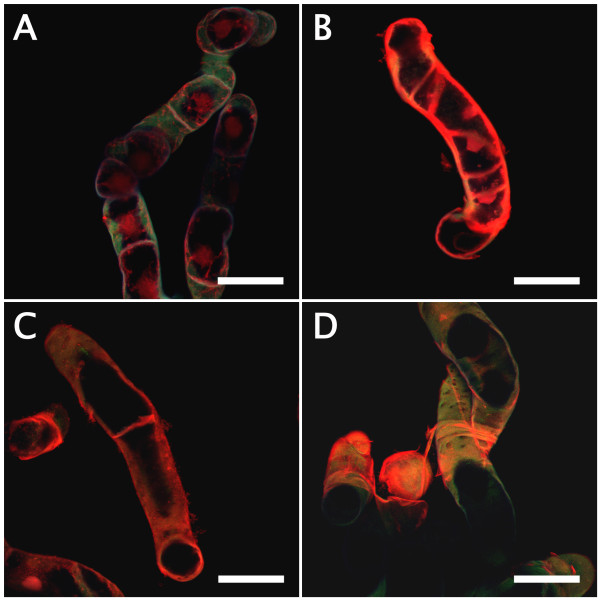
**LSM imaging of suspension cultured bamboo cells stained with safranin.** (**A**) 2-week-old cells under SC conditions. (**B**) and (**C**) 5-week-old cells differentiating into FLEs with cell wall thickening under SC conditions. (**D**) 5-week-old cells differentiating into TEs, with formation of perforations, under SC conditions. Scales = 50 μm.

Bamboo suspension cells proliferated from 2.5% (v/v) sedimented cell volume (SCV) of initial cell volume to 40% (v/v) SCV in subculture conditions (SC), i.e. modified half-strength Murashige and Skoog [15] medium (m1/2MS) supplemented with 3 μM 2,4-dichlorophenoxyacetic acid (2,4-D) in 2 weeks of subculture, then degraded toward a necrosis/xylogenesis stage around 3–5 weeks [13]. We collected subcultured cells weekly and stained them with phloroglucinol-HCl, which is used to identify lignin [16]. As shown in Figure 1A, 4 to 5-week-old cells were stained pale-pink to red. Alcohol-insoluble residues (AIR) and lignin thioglycolate (LTGA) respectively increased up to ca. 65% and 4% of the dry weight in 4-week-old cells and ca. 70% and 10% of the dry weight in 5-week-old cells (Figures 1B and C). Xylogenic features of suspension cells involved in lignification were observed using a laser scanning microscope (LSM) after safranin staining. Compared with 2-week-old cells (Figure 2A) which had a pale-bluish autofluorescent signal in the thin primary cell wall and a strong orange signal in the nucleus, two types of xylogenic cells, elongated fiber-like elements (FLEs) with cell wall thickening (Figures 2B and C) and tracheary elements (TEs) showing formation of perforations in the cell wall (Figure 2D) were observed in 5-week-old cells. Secondary cell walls that developed in these FLEs and TEs were strongly signalized orange by safranin.

### Promotive factors of lignification

**Figure 3:**
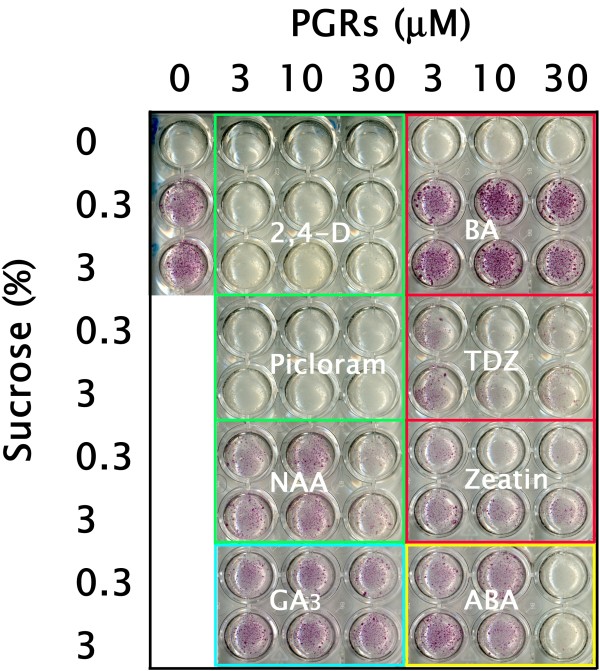
Effects of PGRs on lignification of suspension cultured bamboo cells.

In order to identify promotive factors of lignification in xylogenic suspension cells, liquid m1/2MS medium supplemented with 0, 3, 10, or 30 μM 2,4-D, 4-amino-3,5,6-trichloropyridine-2-carboxylic acid (Picloram), 1-naphthalene acetic acid (NAA), 6-benzyladenine (BA), thidiazuron (TDZ), zeatin, gibberellin A3 (GA_3_), or abscisic acid (ABA) and 0, 0.3, and 3% sucrose were investigated. All cultured cells were collected weekly and stained with phloroglucinol-HCl. Lignification patterns could be seen in 1 week and were clearly distinguishable in 2-week cultures, as shown in Figure 3. In phytohormone-free m1/2MS media containing 0.3 and 3% sucrose, pale-pink to red stains were observed. Without a sucrose supply, no lignification occurred. Compared with auxins such as 2,4-D and Picloram, which were phytohormones that promoted proliferation of bamboo cells (ca 40% and 80% as SCV, respectively), other plant growth regulators (PGRs), such as the cytokinin BA in particular, affected lignification (ca. 25% SCV) but not proliferation. The AIR varied depending on the conditions, and the LTGA increased to ca. 25% of the cells under LG conditions (4A and B).

**Figure 4:**
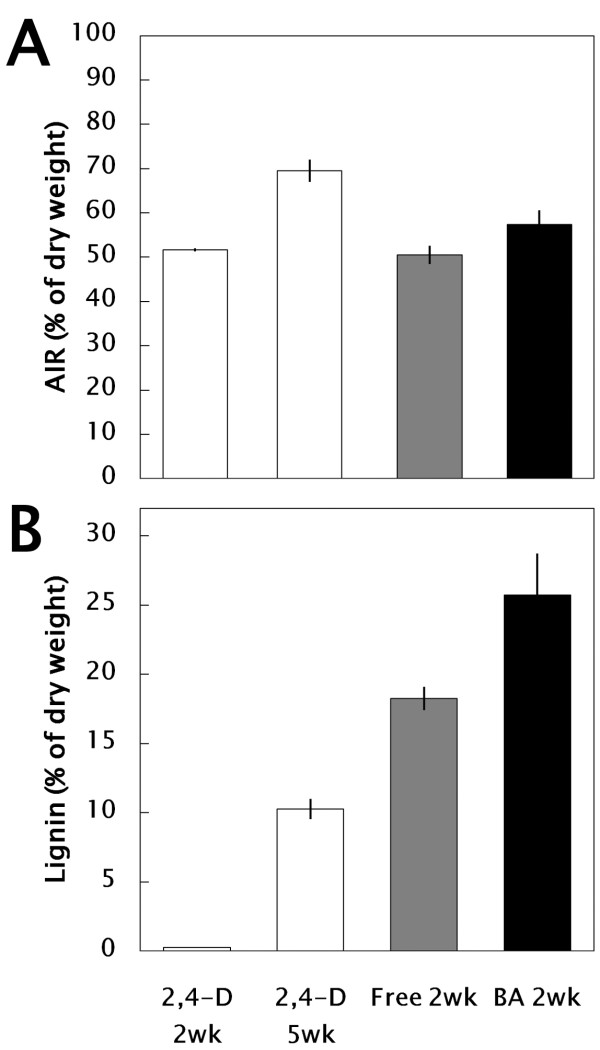
**Lignification capacity of bamboo suspension cultured bamboo cells under SC (2,4-D; m1/2MS + 3** μ**M 2,4-D) and LG (Free; m1/2MS – phytohormones, BA; m1/2MS + 10** μ**M BA) conditions.** (**A**) AIR content. (**B**) lignin content.

**Figure 5:**
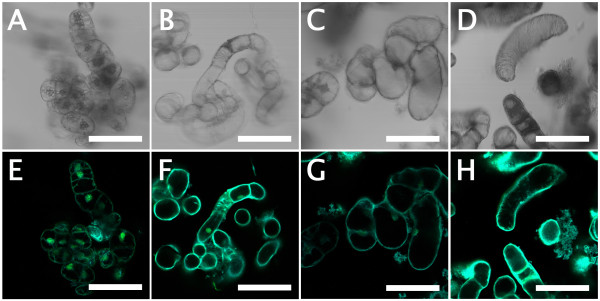
**LSM imaging of suspension cultured bamboo cells stained with Sytox Green.** (**A**) and (**E**) 2-week-old cells under SC conditions. (**B**) and (**F**) 5-week-old cells under SC conditions. (**C**) and (**G**) 2-week-old cells differentiated into FLEs under LG conditions (phytohormone free). (**D**) and (**H**) 2-week-old cells differentiated into TEs under LG conditions in the presence of BA. Scales = 100 μm.

**Figure 6:**
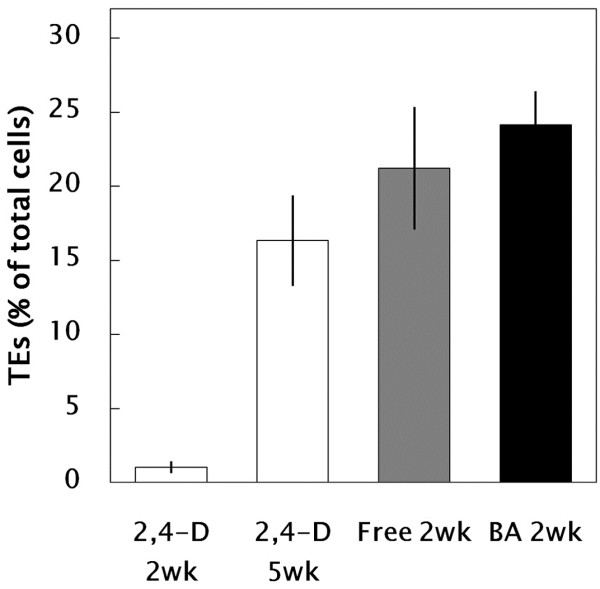
**Effect of culture conditions on differentiation of TEs (2,4-D: m1/2MS + 3** μ**M 2,4-D; Free: m1/2MS – phytohormones; BA: m1/2MS + 10** μ**M BA).** A portion (ca. 20 μl) of suspension cells that contained ca. 50–100 cells in total was mounted on a glass slide and the number of TEs in the sample was counted under a microscope. All data are expressed as mean ± SE (n = 18).

The patterns of cell growth, differentiation and death involved in xylogenesis of suspension cells were observed using LSM with Sytox Green staining (Figure 5). The results supported the observations obtained after safranin staining (see Figure 2). Briefly, LSM imaging with Sytox Green revealed that 2-week-old cells originating from SC conditions had a pale-bluish autofluorescent signal in the primary cell wall and a strong greenish signal in the nucleus, whereas 5-week-old cells originating from SC conditions had a much brighter pale-bluish autofluorescent signal in the secondary cell wall and weaker or no greenish signal in the nucleus. Xylogenic features, like those seen in 5-week-old cells, could be observed frequently in 2-week-old cells originating from LG conditions. The number of TEs with formation of perforations in the cell wall increased to ca. 25% in BA-treated cells (Figure 6).

### Identification of substructures of lignin in bamboo cells

**Figure 7:**
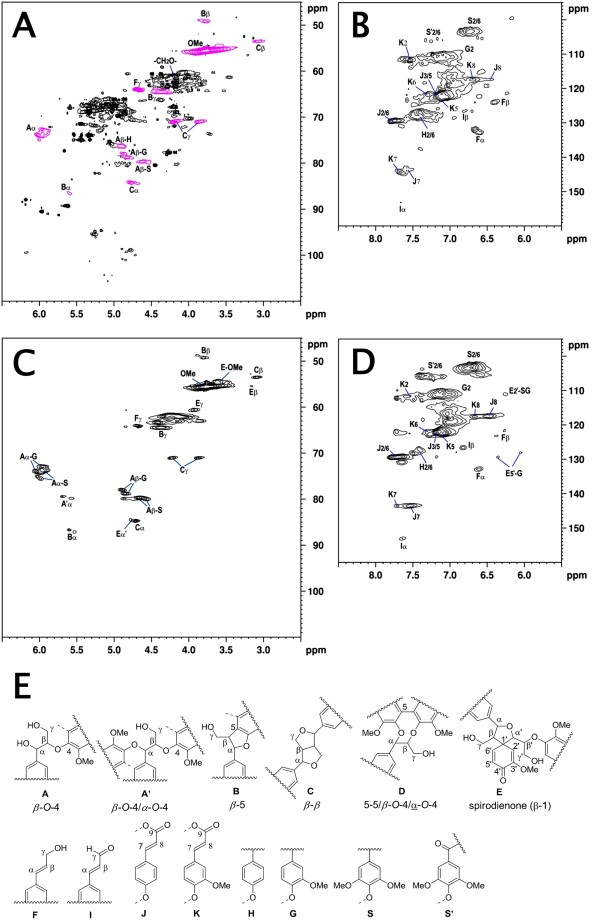
**HSQC NMR spectra of acetylated BCCL and BMWL.** (**A**) aliphatic region of BCCL, (**B**) aromatic region of BCCL, (**C**) aliphatic region of BMWL, (**D**) aromatic region of BMWL, (**E**) substructures of lignin identified in this study.

Heteronuclear single quantum coherence (HSQC) NMR spectra of acetylated cultured bamboo cell lignin (BCCL; Figures 7A and B) and milled bamboo wood lignin (BMWL; Figures 7C and D) were acquired to clarify the lignin components. The substructures of lignin identified in this study are summarized in Figure 7. The main lignin substructures, including β-aryl ether (β-*O*-4, A), phenylcoumaran (β-5, B), resinol (β-β, C) and cinnamyl alcohol (F) were distinct in the aliphatic region of the HSQC spectra (Figure 7A). The aromatic region is shown in Figure 7B. Strong signals for H2/C2, H5/C5, and H6/C6 in guaiacyl (G) units and H2/C2 and H6/C6 in syringyl (S) units were recognized. The signals for H2/C2 and H6/C6 in S units with α-carbonyl groups (S′ units) were very weak. In addition to these signals, relatively strong signals corresponding to *p*-hydroxyphenyl (H) units were visible. Since bamboo lignin is known as a mixed polymer of G and S units with a small amount of H units [17], the relative proportions of H/G/S units estimated by signal intensities are shown in Table 1. The esters of *p*-coumaric acid (J) and ferulic acid (K), which are well known as typical grass lignin-related compounds [18], were also detected.

**Table 1:** Composition of H/G/S lignins in bamboo identified by NMR

**Sample**	**% H**	**% G**	**% S**
BMWL	3.7	52.2	43.8
BCCL	19.1	65.3	15.6

### RT-PCR analysis of xylogenic suspension cells

**Figure 8:**
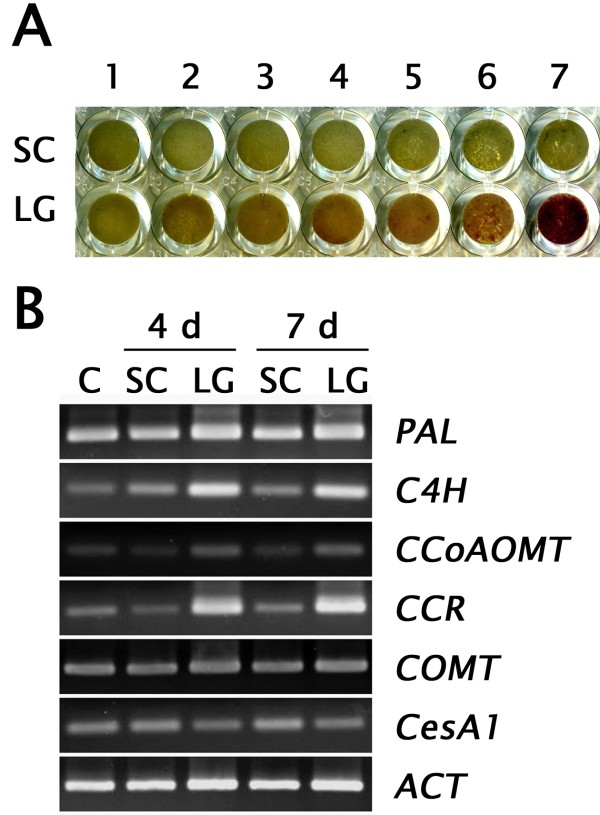
**Transcription profiles of xylogenesis-related genes.** (**A**) Images of phloroglucinol-HCl staining during the 7 days of culturing. (**B**) RT-PCR. C: control (0 d).

First, we collected suspension cells daily and stained them with phloroglucinol-HCl. As shown in Figure 8A, pale-pink to red signals were detectable in 4- to 7-day-old cells cultured under LG conditions, indicating that lignin biosynthesis could be activated in 4–7 days. As expected, relative transcription levels of key genes associated with early stages of lignin biosynthesis, such as *PAL1*, *C4H*, *CCoAOMT*, and *CCR*, were induced under LG conditions (Figure 8B).

## Discussion

The xylogenic culture of *Zinnia* mesophyll cells established by Fukuda and Komamine [19] is one of the most useful systems for investigation of sequential events during differentiation of TEs through physiological, genetic and genomic approaches [20,21]. Due to the fact that xylogenic features of intact bamboo plants are quite complicated, as shown in Additional file 1: Figure S1, we focused on establishing an ideal suspension cell culture model of *P. nigra* for exploring the processes of xylogenesis and lignification.

The role of auxin and sucrose in TE differentiation in tissue cultures of plants such as *Daucus carota, Syringa vulgaris, Glycine maxHelianthus annuus, Hibiscus cannabinus* and *Pisum sativum* was mentioned by Aloni [22]. Since then, the effects of other PGRs have been reported; e.g., cytokinin is effective for extracellular lignin formation in suspension cultures of *Picea abies*[23] and the addition of GA_3_ to xylogenic *Zinnia* cells increases lignin content [24]. In our experiments, cytokinins such as BA, zeatin and TDZ were effective at suppressing proliferation of cells and inducing lignification, although some differences in the intensity of phloroglucinol staining could be seen. A large amount of lignin, up to ca. 25% of the cells as LTGA, was rapidly deposited within 2 weeks under optimized LG conditions (10 μM BA) in the bamboo suspension culture model. We also found that without a sucrose supply, lignification was not promoted at all in the presence of any PGRs. This result correlates well with the sugar-dependent accumulation of phloroglucinol-positive staining and activation of lignin biosynthesis in hypocotyls of *A. thaliana*[25]. Based on these results, it seems that cytokinin and sucrose are very important promotive factors for xylogenic differentiation and lignin biosynthesis in bamboo cells. As hormone and other small molecule interactions during different phases of vascular tissue development have been recently documented using several model plants such as *ArabidopsisPopulus*, and *Zinnia*[26], further analysis allowing understanding of the cross-talk of PGRs in bamboo will be possible using our xylogenic suspension culture model.

The most important finding of this study is that 2-week-old suspension cells are highly lignified, to approximately 25% of the dried cells under LG conditions. This is extremely high compared to other cell culture models such as *Populus*[27] and *Arabidopsis* T87 [28]. Furthermore, the presence of the most common interunit linkages, including β-aryl ether (β-*O*-4, A), phenylcoumaran (β-5, B) and resinol (β-β, C) structures was identified in the BCCL by HSQC NMR. In addition to these common features of lignin, several differences in lignin substructures were also found between the BCCL and the BMWL. H/G/S units also could be seen in the BCCL. These observations suggest the efficacy of our xylogenic cell culture model as a powerful tool for exploring the dynamics of the lignification process of *Phyllostachys* bamboo.

We obtained the following two promising results: (1) we could identify and control two types of xylogenic differentiation, FLEs with cell wall thickening and TEs with formation of perforations in the cell wall and (2) Transcription of xylogenesis-related genes such as *PAL1C4HCCoAOMT*, and *CCR* were induced in a suitable duration of culture under LG conditions. Based on these results, our xylogenic suspension culture model would be useful for detailed characterization of the transcriptional regulation of secondary wall formation and vascular tissue development, e.g., the MYB [29,30] and NAC [31,32] transcription factors of bamboo. Detailed studies for further characterization of physiological and molecular biological events of bamboo cells using metabolomics and next-generation sequencing technologies are now in progress.

## Methods

### Suspension culture

A bamboo (*P. nigra*) suspension culture was established and maintained in subculture conditions (SC), i.e., m1/2MS supplemented with 3 μM 2,4-D, as described earlier [11]. For maintenance subculturing, a portion of liquid suspension cells (2.5% (v/v) SCV) was transferred to fresh m1/2MS medium and placed on a rotary shaker at 100 rpm in the dark at 25°C. The SCV was measured by holding a suspension of the cells for 15 minutes in a 50 ml centrifugation tube graduated in milliliters [13].

### Lignification capacity of bamboo suspension cells

Lignification capacity of bamboo suspension cells under SC conditions was first monitored by a phloroglucinol-HCl reaction [16]. Then, in order to enhance the biosynthesis of materials related to lignification in suspension cells, liquid m1/2MS media supplemented with 0, 3, 10, and 30 μM 2,4-D, Picloram, NAA, BA, TDZ, zeatin, GA_3_, or ABA were prepared. The concentration of sucrose, one important inducer of lignin biosynthesis [25], was varied at 0, 0.3, and 3% (w/v). A portion of liquid suspension cells (2.5–5% (v/v) initial cell density as SCV) was transferred to each set of conditions and cultured for 3 weeks in the dark at 25°C. The cultured cells were collected weekly and stained with phloroglucinol-HCl [16].

### Imaging analyses

Lignified cells detected by a phloroglucinol-HCl reaction were observed under an imaging scanner (PM-850; Epson, Japan). The cultured cells were collected, fixed using 4% (v/v) glutaraldehyde solution in sodium phosphate buffer (0.1 M, pH 7.2), and dehydrated in an ethyl alcohol series. After dehydration, they were stained with 0.01% safranin or 0.5 μM Sytox Green and observed using an LSM 510 META laser scanning microscope (Zeiss, Germany) for characterization of cell growth and death, cell enlargement and cell wall thickening patterns.

Bamboo cells were cultured under both SC and LG conditions as described above and collected at appropriate periods. Total RNA was prepared from the cells with an RNeasy Plant Mini kit (Qiagen, Germany) and amplified by RT-PCR using a SuperScript III First-Strand Synthesis System for RT-PCR (Invitrogen, USA) with gene specific primers (Additional file 2: Table S1). Sequence data for this study can be found in the GenBank data libraries under the following accession numbers: FJ594467 (*PAL1*), FP092384 (*C4H*), FP094113 (*CCoAOMT*), FP098108 (*CCR*), FP094027 (*COMT*), FJ495287 (*CesA1*), and FJ601918 (*Actin*). PCR was performed in a 20 μl reaction mixture containing 2 μl 2 mM dNTPs, 2 μl 10 x Blend Taq buffer, 0.5 unit Blend Taq polymerase (Toyobo, Japan), 1 μl primers (10 μM) and 100 ng template cDNA. Amplification was carried out under the conditions of 30 cycles of denaturation at 96°C for 30 s, annealing at 60°C for 30 s and extension at 72°C for 1 min. The amplified DNAs were detected by ethidium bromide staining after agarose gel electrophoresis.

### Isolation of lignin

Mature bamboo culms (ca. 2 years old) were ground in a microfine grinding mill with an MF 10 cutting/grinding head (IKA Works, USA) and fractionated by sieves. The fractionated milled bamboo (0.15–0.35 mm) was extracted with ethanol-benzene (1:2, v/v) for 6 h and dried under vacuum over P_2_O_5_. The extractive-free milled bamboo (3 g) was further ground in a planetary mono mill P-6 (Fritsch, Germany) under an argon atmosphere for 8 h in a 45-ml ZrO_2_ jar with 18 balls. The milling frequency was 600 rpm. A 15-min pause was introduced after every 30 min of milling to prevent overheating. BMWL was isolated according to the Björkman method [33], and the isolated BMWL was purified by the procedure of Lundquist [34]. The yield of BMWL was 1.4%, based on the extractive-free dry plant material. BCCL was also isolated as described above with a slight modification. Briefly, freeze-dried cultured cells in the presence of10 μM BA were extracted with 80% (v/v) methanol, dried under vacuum, and ball-milled for 8 h. The BCCL was isolated by the Björkman method without any purification. The yield of BCCL was 10.3%. BMWL was also used to construct a calibration curve for LTGA measurement as described below. Both isolated lignins, BMWL and BCCL, were acetylated with pyridine-acetic anhydride for 24 h in the dark at room temperature for NMR analysis.

### LTGA measurement

Lignin was quantitatively assayed by the thioglycolic acid procedure of Bruce and West [35]. Suspension cells of 2 and 5 weeks old under the SC conditions (3 μM 2,4-D); 2 weeks old under LG conditions, i.e., phytohormone-free or cultured with 10 μM BA were collected, freeze-dried and stored in an ultralow-temperature freezer (−80°C) before use. AIR (50 mg), obtained by treatment of freeze-dried cells with 80% (v/v) MeOH for 30 min at 80°C, were placed in a glass screw-cap tube containing 5 ml 2 N HCI and 0.5 ml thioglycolic acid. The sealed tubes were placed in a boiling water bath and shaken initially to hydrate the AIR. After 4 h at 100°C, the tubes were cooled and the contents transferred to polypropylene centrifuge tubes. Following centrifugation at 30,000 *g* for 10 min at room temperature, the supernatant was discarded and the pellet washed once with 5 ml H_2_O. The resulting pellet was resuspended in 5 ml 0.5 N NaOH, sealed with Parafilm, and agitated gently at 25°C for 18 h to extract the LTGA. The samples were centrifuged (30,000 *g*, 10 min) and the supernatant solutions were transferred to conical glass centrifuge tubes. Concentrated HCI (1 ml) was added to each tube and the LTGA was allowed to precipitate at 4°C for 4 h. Following centrifugation in a clinical centrifuge at top speed for 10 min, the orange-brown pellets were dissolved in 10 ml 0.5 N NaOH, and the absorbance at 280 nm was measured.

### NMR spectroscopy

Acetylated BMWL or BCCL (30–40 mg) were dissolved in 0.5 ml DMSO-*d*_6_, and transferred to an NMR sample tube. NMR spectra were recorded on a 500-MHz NMR spectrometer (Avance II, Bruker, Germany) with a 5-mm BBI probe at 300 K using DMSO-*d*_6_ as a solvent. The Bruker standard pulse program hsqcetgpsi2 was used for HSQC experiments [36,37]. Chemical shifts were referenced to DMSO-*d*_6_ (2.50/39.5 ppm).

## Abbreviations

Picloram, 4-amino-3,5,6-trichloropyridine-2-carboxylic acid; 2,4-D, 2,4-dichlorophenoxyacetic acid; NAA, 1-naphthalene acetic acid; BA, 6-benzyladenine; TDZ, Thidiazuron; GA_3_, Gibberellin A3; ABA, Abscisic acid; SCV, Ssedimented cell volume; Subculture conditions, SC conditions; Lignification conditions, LG conditions; FLEs, Fiber-like elements; TEs, Tracheary elements; LSM, Laser scanning microscope; HSQC NMR, Heteronuclear single quantum coherence NMR; AIR, Alcohol-insoluble residues; LTGA, Lignin thioglycolate; BCCL, Bamboo cultured cell lignin; BMWL, Bamboo milled wood lignin; PGR, Plant growth regulator.

## Authors’ contributions

SO designed the experiments, analyzed the data and wrote the manuscript. TN, TK and YK assisted in the work and interpreted data. All authors read and approved the final manuscript.

## Supplementary Material

Additional file 1 **Figure S1.** Lignification capacity of bamboo (*P. nigra*) culms. (**A**) Large numbers of vascular bundles densely distributed toward the outer region of the culm (ca. 1.5-year-old bamboo). Scale = 1 mm. Cross-sections (ca. 15 μm) were cut from the culm and stained with phloroglucinol-HCl. (**B**) Outer region of mature culm. Scale = 50 μm. (**C**) Inner region of mature culm. Scale = 50 μm. Pa: parenchyma cells, Ph: phloem, Sc: sclerenchyma cells, Xy: xylem. Mosaic staining patterns were detected, especially in the outer region of the culm. Two types of staining patterns were observed in parenchyma cells of the ground tissues (pale pink), and in fiber elements such as xylem and sclerenchyma cells of the vascular bundles (red). Click here for file

Additional file 2 **Table S1.** Oligonucleotides used in this study. Click here for file
