# Supplementary figures and images for: A novel xylogenic suspension culture model for exploring lignification in Phyllostachys bamboo
Source: Plant Methods. 2012 Sep 14;8:40. doi: 10.1186/1746-4811-8-40 (PMC3462127; doi:10.1186/1746-4811-8-40)

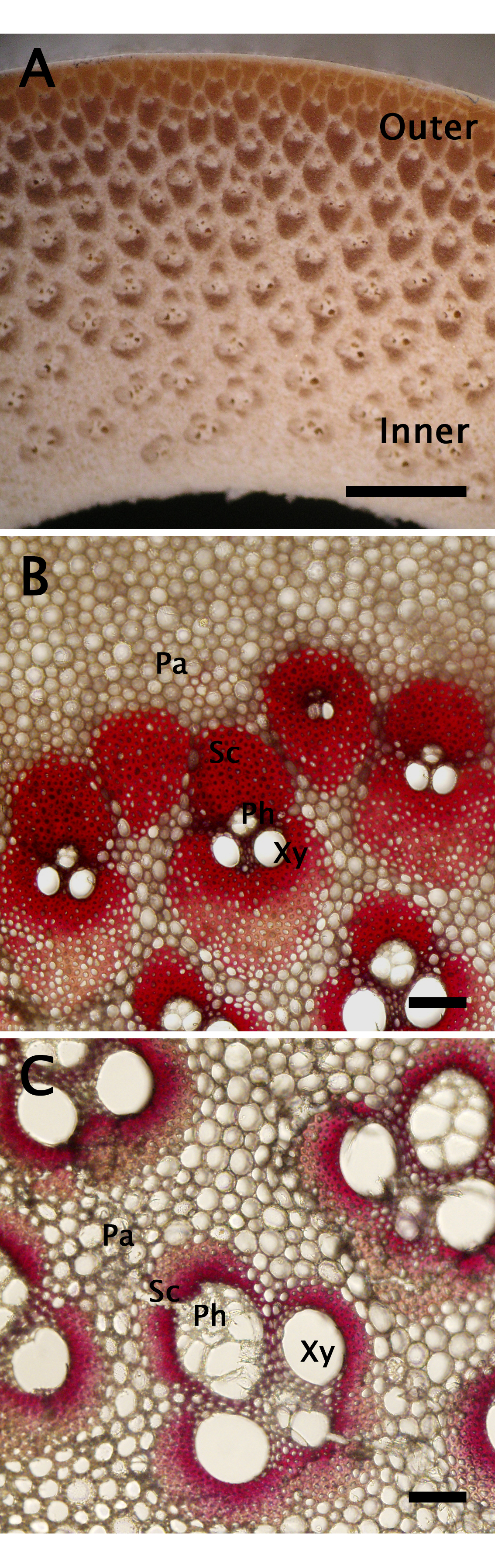

Supplement: Additional file 1 — Figure S1. Lignification capacity of bamboo (P. nigra) culms. (A) Large numbers of vascular bundles densely distributed toward the outer region of the culm (ca. 1.5-year-old bamboo). Scale = 1 mm. Cross-sections (ca. 15 μm) were cut from the culm and stained with phloroglucinol-HCl. (B) Outer region of mature culm. Scale = 50 μm. (C) Inner region of mature culm. Scale = 50 μm. Pa: parenchyma cells, Ph: phloem, Sc: sclerenchyma cells, Xy: xylem. Mosaic staining patterns were detected, especially in the outer region of the culm. Two types of staining patterns were observed in parenchyma cells of the ground tissues (pale pink), and in fiber elements such as xylem and sclerenchyma cells of the vascular bundles (red). [file 1746-4811-8-40-S1.jpeg]
