# Supplementary material for: A novel xylogenic suspension culture model for exploring lignification in Phyllostachys bamboo
Source: Plant Methods. 2012 Sep 14;8:40. doi: 10.1186/1746-4811-8-40 (PMC3462127; doi:10.1186/1746-4811-8-40)
Supplement: Additional file 2 — Table S1. Oligonucleotides used in this study. [file 1746-4811-8-40-S2.doc]

| **Supplemental Table S1.** Oligonucleotides used in this study. | |
| --- | --- |
| Name | Sequence (5’>3’) |
| PAL1-for2 | GTCCGCATCAACACTCTCCT |
| PAL1-rev2 | CTTCAGCTTGTGGGTCAGGT |
| C4H-for1 | GCAGTTCAGCCTCCACATC |
| C4H-rev1 | GCCACCCGCTAGTCAAGTAA |
| CCoAOMT-for1 | CCTTCGTTTGTTACGCTTGG |
| CCoAOMT-rev1 | GAGGGTCTTGATGCTGTCGT |
| CCR-for1 | CACTTTCTCTGCGTTTCTGCT |
| CCR-rev1 | CTTCGGGTCATCTGGGTTC |
| COMT-for2 | CCACTCCATCATCATCACCA |
| COMT-rev2 | AGCTCCTCGAACTCCCTCTC |
| CesA1-for1 | AGGGAGATGAGGAGGAGGAA |
| CesA1-rev1 | GGCGAGAGGATGAAGACAGA |
| ACT-for1 | CCCAAGGCAAACAGAGAGAA |
| ACT-rev2 | AAAAGATGGCTGGAAGAGCA |
